# An investigation into perceived autonomy support, motivation and competence in chronic pain patients in Ireland: A cross-sectional study

**DOI:** 10.1371/journal.pone.0301861

**Published:** 2024-05-06

**Authors:** Kate Sheridan, Siobhan O’Connor, Enda Whyte

**Affiliations:** Centre for Injury Prevention and Performance, School of Health and Human Performance, Dublin City University, Dublin City, Ireland; University of Huelva: Universidad de Huelva, SPAIN

## Abstract

Autonomy supportive healthcare settings are associated with enhanced behaviour change and self-management strategies in individuals living with chronic disease. The level of autonomy support provided by healthcare professionals to individuals living with chronic pain in Ireland is unknown. A cross-sectional study was completed on participants living with chronic pain (>3 months) in Ireland. Participants (n = 389) completed an anonymous survey constructed of patient reported outcome measures relating to autonomy support (HCCQ), motivation (TSRQ), competence in physical activity (PCS), pain interference (BPI) and psychological factors (PHQ-9, GAD-7). Results showed the median HCCQ (*H* = 39.287, *p* < .001), Autonomous Motivation (*H* = 13.568, *p* = 0.019) and PCS (*H* = 30.701, *p* < .001) scores were significantly different when patients received care from different healthcare professionals. There was a negative correlation between PCS and pain severity (r = -0.32, <0.01), pain interference (r = -0.44, p = <0.01), PHQ-9 (r = -0.50, p = <0.01) and GAD-7 (r = -0.34, p = <0.01). This study has identified that perceived healthcare support in Ireland varies according to the healthcare professional leading pain care. Furthermore, higher levels of self-determination were associated with decreased depression and anxiety in individuals with chronic pain. Given the limited number of multidisciplinary team clinics to provide pain management programs, an alternative cost-effective community led solution is required. The results of this study indicate that allied health professionals may be well placed to fill this void. Future research exploring the barriers to providing healthcare supportive settings is required.

## 1. Introduction

Pain and pain-related diseases are the leading cause of disability and disease burden globally [1]. The treatment of chronic pain is complex and requires the integrated consideration of biological, psychological and social factors [2,3]. Multidisciplinary Team (MDT) clinics are advocated as best practice to support the complex treatment of chronic pain [4–6]. MDT clinics promote an individual’s ability to self-manage their pain through education and behaviour change techniques [4,5].

In Ireland, despite a chronic pain prevalence of 35% [7], pain services are under-resourced [8]. Sixteen public pain management clinics offer interventional pain therapies across the nation of Ireland, but only five clinics offer full MDT pain management services [9]. Consequently, in many cases, the delivery of pain management services and the development of self-management skills in Ireland is conducted by individual clinicians rather than an MDT.

Self-management has been described as a person’s capacity to manage their symptoms and the physical and psychosocial impacts of pain on their daily lives [10]. In order to successfully self-manage chronic pain, behaviour change interventions should be employed by healthcare professionals [11]. Self-Determination Theory (SDT) encourages the development of enhanced self-management skills by supporting intrinsic motivation and developing behaviour change [12–15]. According to SDT, behaviour change can be enhanced if a person’s autonomy (feeling free to engage in a behaviour), competence (feeling effective to engage in a behaviour) and relatedness (feeling cared for and valued) are optimised in healthcare settings [16,17].

Autonomy supportive healthcare settings have been associated with enhanced biological and psychosocial outcomes in individuals living with chronic disease [18]. Specific to chronic pain, enhanced levels of autonomy and competence have been associated with decreased disability levels [19]. Hence, autonomy supportive healthcare settings that support health behaviours are beneficial and desirable in chronic pain conditions. As healthcare professionals in Ireland generally do not have the support of MDT clinics in providing care, they are required to support autonomy and competence in self-management skills (such as physical activity) independently.

The level of autonomy support provided by individual healthcare professionals is currently unknown in Ireland. In order to understand patient experience, an investigation into the existing levels of autonomy support in Irish healthcare settings is required. Therefore, this study aimed to examine the components of SDT (autonomy support, motivation and competence in physical activity) of individuals living with chronic pain in Ireland. Secondly, this study aimed to explore differences in perceived autonomy support, motivation and competence in physical activity in individuals undergoing treatment with different Irish healthcare professionals. Finally, the association between pain interference, psychological wellbeing and the components of self-determination theory (autonomy, motivation and competence in physical activity) among chronic pain sufferers in Ireland were explored.

## 2. Materials and methods

### 2.1 Participants and procedures

Adult participants living with chronic pain (pain > 3 months) in Ireland were eligible for study inclusion. To achieve a confidence level of 95%, the required sample size was determined as 385 participants [online software www.raosoft.com]. Sample size was calculated based on a prevalence of chronic pain in Ireland of 35% [7] and a population in Ireland in 2022 of 5.15 million [20]. Ethical approval for this study was granted by Dublin City University research ethics committee (DCUREC/2022/082). Informed consent was retrieved electronically at the beginning of the online survey. Completion of the study’s informed consent form was required in order for participants to progress to the anonymised survey.

Participants were recruited from 1^st^ August 2022 to 30^th^ of October 2022 with survey reminders sent at 2-week intervals. The study was promoted a) on social media (Twitter, Facebook, Instagram) by two chronic pain advocate groups, b) by poster in Pain Management, Orthopaedic and Neurosurgical Clinic waiting rooms and c) in one medical exercise clinic. Participants completed an anonymous online survey (mean completion time 13 minutes SD = 1.8) using the Qualtrics© online survey platform [21].

### 2.2 Outcome measures

To commence the survey participants were asked to disclose if they lived with chronic pain. Chronic pain was defined in the introduction of the survey as pain lasting 3 months or longer [22]. If participants self-reported not having chronic pain the survey ended. If they self-reported living with pain lasting three or more months they proceeded to section one. Section one of the survey recorded demographic information including gender, age, county, diagnosis and years lived with pain. Section two of the data collection protocol included reliable and validated questionnaires in the following order, Health Care Climate Questionnaire (HCCQ) [23], Brief Pain Interference (BPI) [24], Treatment Self Regulating Questionnaire (TSRQ) [23], Perceived Competence Scale (PCS) [23], Patient Health Questionnaire-9 (PHQ-9) [25] and the General Anxiety Disorder-7 (GAD-7) [26].

#### 2.3.1 Health care climate questionnaire

Perceived autonomy support was assessed using the 15-item Health Care Climate Questionnaire (HCCQ) [23]. The HCCQ identifies the participant’s perceptions of which their primary healthcare professional is autonomy supportive. The primary healthcare professional was defined in the survey as the person the participant considered to have the most influence on their treatment and pain management. Responses were given on a 7-point Likert scale (1 strongly disagree to 7 strongly agree). Participant scores were averaged, with higher mean scores demonstrating higher perceived autonomy support. The scale had a high level of internal consistency (Cronbach’s alpha = 0.97).

#### 2.3.2 Treatment self regulating questionnaire

Motivation towards regular physical activity was assessed using the 15-item Treatment Self Regulating Questionnaire (TSRQ) [23]. Responses are given using a seven-point Likert scale (1 not at all true to 7 very true). The TRSQ consists of three subscales, the 6-item autonomous motivation (the patient is internally motivated), the 6-item controlled motivation [the patient is externally motivated] and the 3-item amotivation scale (the patient is not motivated). The autonomous motivation subscale consists of items # 1, 3, 6, 8, 11, & 13; the controlled motivation subscale consists of items # 2, 4, 7, 9, 12, & 14; and the amotivation subscale consists of items # 5, 10, & 15. Participant scores for each subscale were averaged individually, with higher scores demonstrating higher levels of motivation or amotivation. The combined scale (Cronbach’s alpha = 0.76) along with the subscales autonomous motivation (Cronbach’s alpha = 0.91) and controlled motivation (Cronbach’s alpha = 0.76) demonstrated high internal consistency. The subscale amotivation was considered optimal (mean inter-item correlation = 0.26) [27].

#### 2.3.3 Perceived competence scale

Perceived competence towards regular physical activity was assessed using the 4-item Perceived Competence Scale (PCS) [23]. Responses are given using a seven-point Likert scale (1 not at all true to 7 very true). An individual’s score was calculated by averaging the total score of the 4-items. The scale had a high level of internal consistency (Cronbach’s Alpha = 0.96).

#### 2.3.4 Brief pain inventory

Pain intensity and pain interference scores were assessed using the Brief Pain Inventory (BPI) [24]. Pain intensity was measured with four items (worst, least, average and current pain intensity). Pain interference was measured with seven items, including general activity, mood, walking, work, relations with others, sleep and enjoyment of life. Both pain intensity and pain interference responses were gathered on a 0–10 scale with 10 indicating the worst imaginable pain and complete pain interference. An individual’s pain intensity score was calculated by averaging the total score of the 4-items. The pain interference score was calculated by averaging the total score of the 7-items. Both pain severity (Cronbach’s alpha = 0.86) and pain interference (Cronbach’s alpha = 0.91) scales had a high level of internal consistency.

#### 2.3.5 Patient health questionnaire

The presence and severity of depression was assessed using the Patient Health Questionnaire-9 (PHQ-9) [25]. The PHQ-9 consists of 9-items relating to depression that correspond to the Diagnostic and Statistical Manual of Mental Disorders [28]. Responses are scored from 0 (not at all) to 3 (nearly every day) with a total score of 27 possible. In chronic physical health conditions, a cut off score ≥10 has been utilised for depression identification [29]. The scale demonstrated a high level of internal consistency reporting a Cronbach’s alpha of 0.87.

#### 2.3.6 Generalised anxiety disorder

The presence of anxiety was assessed using the Generalised Anxiety Disorder (Gad-7) [26]. Responses are scored 0 (not at all) to 3 (nearly every day) with a total score of 21 possible. When screening for an anxiety disorder a recommended cut-off point of ≥10 has been utilised for anxiety identification [30]. The scale had a high level of internal consistency (Cronbach’s alpha = 0.92).

### 2.4 Statistical analysis

Statistical Analysis was performed with IBM SPSS statistics version 27 [31]. Descriptive and analytical statistics were analysed to evaluate mean and the standard deviation of the HCCQ, BPI, TSRQ and PCS scores. Statistical analysis was completed to determine if there were differences in HCCQ, TSRQ and PCS between six groups of healthcare workers, “General Practitioner” (GP) (n = 160), “Rheumatologist” (n = 49), “Pain Management Consultant” (n = 50), “Neurologist” (n = 39), “Allied Healthcare Professionals’’ (AHP) (n = 53) and “Other Medical Professionals” (n = 31). Complementary therapists were excluded from group analysis due to insufficient data collection in this group (n = 4). The KolmogorovSmirnov test was used to test for normal distribution. Data did not fulfil requirements for parametric testing; therefore, differences between groups were assessed with the Kruskal-Wallis H-test. Dunn’s test was then used to complete pairwise comparisons with a Bonferroni correction for multiple comparisons [32]. Effect size was classified as 0.1 = small effect, 0.3 = medium effect and 0.5 = large effect [33]. Spearman correlations coefficients were completed to explore the association between BPI, GAD-7, PHQ-9 and HCCQ, TSRQ and PCS scores. Correlation coefficients were classified as low (0.1 and 0.29) moderate (0.3 and 0.49) and high (>0.5) high [34]. Statistical significance was set at *p* < .05.

## 3 Results

### 3.1 Descriptive statistics

A total of 681 participants opened the online survey. Insufficient responses [completing demographic information only] were removed (n = 292). Participants (female n = 321, male n = 65) had a mean age of 48.7 SD = 13.6 (range 18–83) years and reported 11.69 SD = 10.4 (range 0.3–58) years of pain. Participants were recruited from the four provinces of Ireland, Leinster (n = 240), Munster (n = 101), Connacht (n = 29), Ulster (n = 12). Participant demographics are summarised in Table 1. Participants identified a total of 17 healthcare professionals as their primary healthcare professionals (Table 2). Participants reported a mean pain severity of 4.5 SD = 1.9 (range 0–10) and mean pain interference of 5.4 SD = 2.5 (range 0–10). Current treatment strategies reportedly relieved pain by a mean of 41% SD = 27.0 (0–100). Mean PHQ-9 scores were 10.6 SD = 6.4 (range 0–27) and mean GAD-7 scores were 6.9 SD = 5.7 (range 0–21).

**Table 1:** Demographic characteristics of the sample.

Characteristic	N	%
Gender	Female (321)	82.5
	Male (65)	16.7
	Non-Binary (2)	0.5
	Transgender (1)	0.3
Chronic pain conditions	One condition (192)	49
	Two conditions (89)	23
	Three or more conditions (102)	26
Current Treatments	Pharmacology (366)	65
	Conservative Therapy (366)	6
	Pharmacology and Conservative Therapy (366)	12
	None (366)	17

N = number, % = percentage.

**Table 2:** Primary healthcare professionals of persons living with chronic pain.

Healthcare Professional Groups		N	%
General Practitioner		160	41.1%
Pain Management Consultant		50	12.9%
Rheumatologist		49	12.6%
Neurologist		39	10%
Allied Health Professionals	Physiotherapist	48	12.3%
	Athletic Therapist	3	.8%
	Occupational Therapist	2	.5%
Other Medical Health Professionals	Orthopaedic Consultant	12	3.1%
	Neurosurgeon	4	1%
	Gynaecologist	3	.8%
	Nurse	3	.8%
	Oncologist	2	.5%
	Infectious Disease Consultant	2	.5%
	Endocrinologist	2	.5%
	Respiratory Consultant	2	.5%
	General Medicine Consultant	1	.3%
Complimentary Therapists	Massage Therapist	4	1%

N = number, % = percentage.

### 3.2 Autonomy support, motivation, and competence levels

The levels of perceived autonomy support, motivation and competence of individuals living with chronic pain in Ireland are detailed in Table 3. Responses are given using a seven-point Likert scale (1 not at all true to 7 very true) with higher scores demonstrating higher levels of autonomy, motivation and competence.

**Table 3:** Autonomy support, motivation and competence levels.

Outcome Measure (n)	Subscales (n)	Median	Mean ±SD (Range)
HCCQ (389)		4.8	4.6 ±1.7 (0.8–7)
TSRQ	Autonomous Motivation (352)	5.3	5.2 ±1.5 (0–7)
	Controlled Motivation (353)	2.6	2.8 ±1.2 (0–7)
	Amotivation (353)	2	2.2 ±1.2 (0–7)
PCS (351)		3.7	3.6 ±1.9 (0–7)

Note: HCCQ (Healthcare Climate Questionnaire), TSRQ (Treatment Self-Regulation Questionnaire), PCS (Perceived Competence Scale), SD (Standard Deviation).

### 3.3 Individual healthcare professional groups

#### 3.3.1 Healthcare climate questionnaire

A statistically significant difference between healthcare professional groups was observed for HCCQ scores (*H*(5) = 39.287, *p* < .001, η^2^ = .11) (Table 4). Post hoc analysis revealed statistically significant differences with a medium effect size between AHPs (Md = 6.2) and GPs (Md = 4.3), (p = < .001, *r* = 0.4), AHPs and Neurologists (Md = 4.3) (*p* = 0.002, *r* = 0.41), AHPs and Other Medical Health Professionals (Md = 5.3) (*p* = 0.022, *r* = 0.4) and a small effect size between AHPs and Rheumatologists (Md = 5.2) (*p* = 0.04, *r* = 0.28). No statistical differences were observed between any other group combinations.

**Table 4:** Median outcomes of healthcare professional groups.

	GP	Pain Consultant	Rheumatologist	Neurologist	AHP	Other	P-value
**HCCQ**	4.3	5.5	5.2	4.3	6.2	5.2	<0.01
**TSRQ**							
Autonomous motivation	5.2	5.2	5.2	4.8	6	5.5	0.01
Controlled motivation	2.5	3	2.5	2.8	2.8	2.5	0.21
Amotivation	2	2	2	2	2	1.8	0.87
**PCS**	3.3	3.5	3.3	2	5	4.6	<0.01

Note: HCCQ [Health Climate Questionnaire], TSRQ [Treatment Self-Regulation Questionnaire], PCS [Perceived Competence Score].

#### 3.3.2 Treatment self-regulation questionnaire

For autonomous motivation scores, a statistically significant difference between healthcare professional groups was observed (*H*(5) = 13.568, *p* = 0.019, η^2^ = .05). Post hoc analysis revealed statistically significant medium effect size in median autonomous motivation scores between AHPs (Md = 6) and Neurologists (Md = 4.8) (*p* = .011, *r* = 0.36). No statistically significant differences were observed between groups for controlled motivation scores (*H*(5) = 7048, *p* = .217) or amotivation scores (*H*(5) = 1.844, *p* = .87).

#### 3.3.3 Perceived competence scale

A statistically significant difference between healthcare professional groups was observed for PCS scores (*H*(5) = 30.701, *p* < .001, η^2^ = .094). Post hoc analysis revealed a large effect size between AHPs (Md = 5) and Neurologists (Md = 2) (*p* = .000, *r* = 0.51) and a medium effect size between AHPs and GPs (Md = 3.3) (*p* = 0.000, *r* = 0.32), AHPs and Rheumatologists (Md = 3.3) (*p* = .005, *r* = 0.36), AHPs and Pain Management Consultants (Md = 3.5) (*p* = 0.01, *r* = 0.31) and Other Health Professionals (Md = 4.6) and Neurologists (Md = 2) (*p* = .05, *r* = 0.4).

### 3.4 Correlations

There was negative correlation between HCCQ and both PHQ (r = -0.18, p = <0.01) and GAD-7 (r = -0.11, p = 0.02) (Table 5). There was a negative correlation between autonomous motivation and PHQ (r = -0.18, p = 0.01) and a positive correlation between controlled motivation and PHQ (r = 0.14, p = 0.01) and GAD-7 (r = 0.24, p = <0.01). Amotivation was positively correlated to both pain interference (r = 0.10, p = 0.04) and GAD-7 (r = 0.11, p = 0.02). There was a negative correlation between PCS and pain severity (r = -0.32, <0.01), pain interference (r = -0.44, p = <0.01), PHQ-9 (r = -0.50, p = <0.01) and GAD-7 (r = -0.34, p = <0.01).

**Table 5:** Correlation coefficient between autonomy support, motivation, competence, pain interference, depression and anxiety.

	Pain Severity		Pain Interference		PHQ-9		GAD-7	
	*r*	*p*	*r*	*p*	*r*	*p*	*r*	*P*
HCCQ	-0.07	0.13	-0.09	0.06	-0.18	<0.01	-0.11	0.02
Autonomous Motivation	-0.03	0.51	-0.09	0.06	-0.18	0.01	-0.09	0.09
Controlled Motivation	-0.02	0.67	0.06	0.21	0.14	0.01	0.24	<0.01
Amotivation	0.10	0.05	0.10	0.04	0.05	0.31	0.11	0.02
PCS	-0.32	<0.01	-0.44	<0.01	-0.50	<0.01	-0.34	<0.01

Note: HCCQ (Healthcare Climate Questionnaire), PCS (Perceived Competence Scale), PHQ-9 (Patient Health Questionnaire), GAD-7 (General Anxiety Disorder).

## 4. Discussion

This is the first study of its kind investigating perceived autonomy support, motivation and competence to engage in physical activity in a chronic pain population in Ireland. The recorded level of autonomy support in chronic pain patients in Ireland is lower than autonomy support values reported by primary care patients in Europe [35]. Furthermore, autonomy support levels reported in this study are lower than those reported in other chronic conditions including obesity [23], bipolar disease [36] and melanoma [37]. Similarly, the study’s participant’s competence to engage in physical activity was notably lower than competence to engage in physical activity reported in primary care patients [38]. Despite these findings, it is encouraging, that the results of this study indicate that persons living with chronic pain in Ireland are autonomously motivated. In the context of healthcare, autonomous motivation has been related with enhanced self-management behaviour [17,19,23].

This study reported differences in autonomy support from different healthcare professionals as perceived by individuals with chronic pain with significantly higher levels of autonomy support reported when an AHP was identified as the primary healthcare provider. In contrast to the high HCCQ scores for AHPs found in this study, lower HCCQ scores for AHPs in Ireland have previously been reported [39]. The number of clinical interactions may contribute to the difference in observed autonomy support between AHPs in these two studies as the lower HCCQ scores were recorded after a single AHP patient interaction in an outpatient physiotherapy department [39]. With no limitations to healthcare appointments in the current study, increased clinician patient interactions may have provided a greater opportunity for perceived autonomy support from AHPs.

The current study found that participants living with chronic pain reported the lowest levels of perceived autonomy support from both GPs and neurologists. Although no comparison scores for neurologists are available, similar HCCQ scores have been reported for GPs treating chronic disease in the Netherlands [40]. Although the importance of supporting autonomy in populations living with chronic pain has been established [41], both the environmental context and organisational barriers [time and workload] may negatively affect the implementation of an autonomy supportive setting [42]. Specific to Ireland, GPs and consultants working with patients in chronic pain services are heavily affected by both policy systems and patient factors [43]. The standard GP consultation time is 15 minutes [44], at least half the time available to an AHP. Thus, time and workload could vary widely between different healthcare professionals influencing the results observed in this study.

Similar to perceived autonomy support, individuals with chronic pain treated by AHPs demonstrated the highest scores of autonomous motivation and competence in physical activity, compared to the other groups of healthcare professionals. Although no comparative research specific to chronic pain is available, previous research in both post-surgical rehabilitation [45] and cardiac rehabilitation [46] show similar autonomous motivation scores in healthcare environments led by AHPs. As AHPs are well placed to educate and support physical activity behaviour, it is unsurprising this group demonstrated the highest competence score of all healthcare professionals. Low levels of autonomous motivation and competence in physical activity scores were reported by patients under the care of neurologists, GPs and rheumatologists. Given the limited availability to MDT pain clinics in Ireland [9], many patients receive treatment from a medical professional alone without regular access to an AHP for their pain condition. Consequently, these results are of concern as physical activity is indicated in chronic pain rehabilitation and should be recommended to all persons attending chronic pain services [47]. The individual nature of pain means that there is no specific exercise dose and intensity associated with it’s treatment, thereby complicating the prescription of exercise for medical healthcare professionals [48]. Although medical healthcare professionals may advise patients of the importance of physical activity, healthcare workers require the skill set and time to encourage behaviour change as well as simply imparting knowledge [49]. Future research should explore the specific barriers and facilitators to healthcare professionals supporting patients to achieve higher levels of competency in self-management behaviours such as physical activity.

The final aim of this study was to explore the association between pain interference, anxiety, depression and the components of self-determination theory (autonomy, motivation and competence). Similar to previous research [18], it was observed that higher levels of autonomy support, autonomous motivation and competence were associated with decreased anxiety and depression. As chronic pain patients record high levels of both depression [7] and anxiety [50], it is imperative that any future interventions using self-determination theory report not only improvements in self-management behaviours but also improvements in psychological health. Unexpectedly, in contrast to previous chronic pain research [51], the current study observed no relationship between perceived autonomy support or motivation and pain severity and pain interference. As this study is the first to investigate perceived autonomy, motivation and competence in a diverse Irish chronic pain population, further research is required to explore this relationship further.

### 4.1 Implications to practice

Healthcare professional’s support is required in order to assist chronic pain patients to learn how to self-manage their condition [52]. The results of this study indicate that under the care of some healthcare professional groups patients do not feel strongly supported, and this could potentially affect the development of self-management behaviours. Further research investigating pain services and self-management behaviour in Ireland is warranted. Firstly, an investigation into the specific barriers to autonomy support, motivation and competence in clinical groups with the lowest outcomes scores in chronic pain services should be completed. Secondly, research investigating the individual elements of the self-determination theory taxonomy may assist healthcare professionals working in varying infrastructures to identify the strategies that they can adopt to implement an autonomy supportive setting in their clinical context [53]. As well as supporting clinicians to develop autonomy supportive settings, a further solution to promoting self-determined behaviour is to improve service pathways to AHPs. The results of this study indicate that AHPs are well placed to deliver autonomy supportive healthcare that encourage self-management behaviours.

### 4.2 Limitations

Despite the wide use of SDT outcome measures in health-related research, neither a minimally-important difference or an established cut-off score for the HCCQ, TSRQ and PCS have been established or reported in previous research. Causal relationships among the study variables were not possible in this study. This study recruited diverse participants with a variety of chronic pain conditions and as such did not capture the nuances of a specific chronic pain condition. However, the participant demographics and related variables [e.g., psychological wellbeing, and co-morbidities] were similar to previous pain research completed in Ireland [7] and therefore represented the chronic pain population in Ireland. By adopting a convenience sample, participants self-selected to complete the survey, resulting in selection bias. This study did not record the number of treatment sessions that patients completed with healthcare professionals. It is possible that increased treatment sessions will have related to an increased therapeutic alliance and influence on autonomous support. The authors acknowledge that differences found between groups could also result from confounding variables, such as specific pain conditions, participant age, gender, comorbid health conditions, income level, employment, level of education and health related services which were not investigated as part of the survey. Lastly, the limitations of self-reported data in relation to healthcare research are acknowledged. We acknowledge the tendency for participants to provide what they believe are socially acceptable answers, however, it is hoped the anonymous nature of this study minimised this limitation.

## 5. Conclusion

This is the first study to report perceived autonomy support, motivation and competence to engage in physical activity in people living with chronic pain in Ireland. Perceived healthcare support in Ireland varies according to the healthcare professional leading pain care. Given the shortfall of MDT clinics to support the development of self-management behaviours in Ireland, an alternative cost-effective community led solution is required. The results of this study indicate that AHPs may be well placed to support patients to develop self-determined behaviour and thus enhanced self-management of their pain condition. Future research to explore the barriers to providing healthcare supportive settings from the perspective of both clinicians and person’s living with chronic pain are required.

## Supporting information

S1 Data Data used for analysis. (XLSX)
